# Supplementary material for: An Evaluation of Serum miRNA in Renal Cell Carcinoma: A Systematic Review
Source: Cancers (Basel). 2025 Feb 26;17(5):816. doi: 10.3390/cancers17050816 (PMC11898939; doi:10.3390/cancers17050816)
Supplement: Supplementary file 1 [file cancers-17-00816-s001.zip › cancers-3454957-supplementary.pdf]

Supplementary Table S1: PRISMA (Preferred Reporting Items for Systematic Reviews and Meta-Analyses) checklist 2020.

Section and Topic	Item #	Checklist item	Location where item is reported (pages)
**TITLE**			
Title	1	Identify the report as a systematic review.	1
**ABSTRACT**			
Abstract	2	See the PRISMA 2020 for Abstracts checklist.	1-2
**INTRODUCTION**			
Rationale	3	Describe the rationale for the review in the context of existing knowledge.	2
Objectives	4	Provide an explicit statement of the objective(s) or question(s) the review addresses.	3
**METHODS**			
Eligibility criteria	5	Specify the inclusion and exclusion criteria for the review and how studies were grouped for the syntheses.	3
Information sources	6	Specify all databases, registers, websites, organisations, reference lists and other sources searched or consulted to identify studies. Specify the date when each source was last searched or consulted.	3
Search strategy	7	Present the full search strategies for all databases, registers and websites, including any filters and limits used.	3
Selection process	8	Specify the methods used to decide whether a study met the inclusion criteria of the review, including how many reviewers screened each record and each report retrieved, whether they worked independently, and if applicable, details of automation tools used in the process.	3
Data collection process	9	Specify the methods used to collect data from reports, including how many reviewers collected data from each report, whether they worked independently, any processes for obtaining or confirming data from study investigators, and if applicable, details of automation tools used in the process.	4
Data items	10a	List and define all outcomes for which data were sought. Specify whether all results that were compatible with each outcome domain in each study were sought (e.g. for all measures, time points, analyses), and if not, the methods used to decide which results to collect.	4
	10b	List and define all other variables for which data were sought (e.g. participant and intervention characteristics, funding sources). Describe any assumptions made about any missing or unclear information.	-
Study risk of bias assessment	11	Specify the methods used to assess risk of bias in the included studies, including details of the tool(s) used, how many reviewers assessed each study and whether they worked independently, and if applicable, details of automation tools used in the process.	4
Effect measures	12	Specify for each outcome the effect measure(s) (e.g. risk ratio, mean difference) used in the synthesis or presentation of results.	4
Synthesis methods	13a	Describe the processes used to decide which studies were eligible for each synthesis (e.g. tabulating the study intervention characteristics and comparing against the planned groups for each synthesis (item #5)).	-
	13b	Describe any methods required to prepare the data for presentation or synthesis, such as handling of missing summary statistics, or data conversions.	-
	13c	Describe any methods used to tabulate or visually display results of individual studies and syntheses.	-
	13d	Describe any methods used to synthesize results and provide a rationale for the choice(s). If meta-analysis was performed, describe the model(s), method(s) to identify the presence and extent of statistical heterogeneity, and software package(s) used.	4

Section and Topic	Item #	Checklist item	Location where item is reported (pages)
	13e	Describe any methods used to explore possible causes of heterogeneity among study results (e.g. subgroup analysis, meta-regression).	4
	13f	Describe any sensitivity analyses conducted to assess robustness of the synthesized results.	-
Reporting bias assessment	14	Describe any methods used to assess risk of bias due to missing results in a synthesis (arising from reporting biases).	4
Certainty assessment	15	Describe any methods used to assess certainty (or confidence) in the body of evidence for an outcome.	4
**RESULTS**			
Study selection	16a	Describe the results of the search and selection process, from the number of records identified in the search to the number of studies included in the review, ideally using a flow diagram.	4, 7
	16b	Cite studies that might appear to meet the inclusion criteria, but which were excluded, and explain why they were excluded.	4
Study characteristics	17	Cite each included study and present its characteristics.	5
Risk of bias in studies	18	Present assessments of risk of bias for each included study.	5-6, 11-12
Results of individual studies	19	For all outcomes, present, for each study: (a) summary statistics for each group (where appropriate) and (b) an effect estimate and its precision (e.g. confidence/credible interval), ideally using structured tables or plots.	7-10
Results of syntheses	20a	For each synthesis, briefly summarise the characteristics and risk of bias among contributing studies.	7-10
	20b	Present results of all statistical syntheses conducted. If meta-analysis was done, present for each the summary estimate and its precision (e.g. confidence/credible interval) and measures of statistical heterogeneity. If comparing groups, describe the direction of the effect.	7-10
	20c	Present results of all investigations of possible causes of heterogeneity among study results.	7-10
	20d	Present results of all sensitivity analyses conducted to assess the robustness of the synthesized results.	7
Reporting biases	21	Present assessments of risk of bias due to missing results (arising from reporting biases) for each synthesis assessed.	11
Certainty of evidence	22	Present assessments of certainty (or confidence) in the body of evidence for each outcome assessed.	6, 11-12
**DISCUSSION**			
Discussion	23a	Provide a general interpretation of the results in the context of other evidence.	13
	23b	Discuss any limitations of the evidence included in the review.	14
	23c	Discuss any limitations of the review processes used.	14
	23d	Discuss implications of the results for practice, policy, and future research.	14
**OTHER INFORMATION**			
Registration and protocol	24a	Provide registration information for the review, including register name and registration number, or state that the review was not registered.	3
	24b	Indicate where the review protocol can be accessed, or state that a protocol was not prepared.	3

Section and Topic	Item #	Checklist item	Location where item is reported (pages)
	24c	Describe and explain any amendments to information provided at registration or in the protocol.	-
Support	25	Describe sources of financial or non-financial support for the review, and the role of the funders or sponsors in the review.	15
Competing interests	26	Declare any competing interests of review authors.	-
Availability of data, code and other materials	27	Report which of the following are publicly available and where they can be found: template data collection forms; data extracted from included studies; data used for all analyses; analytic code; any other materials used in the review.	-

Supplementary Table S2: SWiM (Synthesis without metanalysis) checklist.

**SWiM is intended to complement and be used as an extension to PRISMA**			
**SWiM reporting item**	**Item description**	**Page in manuscript where item is reported**	**Other**
Methods			
**1** Grouping studies for synthesis	1a) Provide a description of, and rationale for, the groups used in the synthesis (e.g., groupings of populations, interventions, outcomes, study design)	-	-
	1b) Detail and provide rationale for any changes made subsequent to the protocol in the groups used in the synthesis	-	-
**2** Describe the standardised metric and transformation methods used	Describe the standardised metric for each outcome. Explain why the metric(s) was chosen, and describe any methods used to transform the intervention effects, as reported in the study, to the standardised metric, citing any methodological guidance consulted	3	-
**3** Describe the synthesis methods	Describe and justify the methods used to synthesise the effects for each outcome when it was not possible to undertake a meta-analysis of effect estimates	4	-
**4** Criteria used to prioritise results for summary and synthesis	Where applicable, provide the criteria used, with supporting justification, to select the particular studies, or a particular study, for the main synthesis or to draw conclusions from the synthesis (e.g., based on study design, risk of bias assessments, directness in relation to the review question)	4	-

**SWiM reporting item**	**Item description**	**Page in manuscript where item is reported**	**-**
**5** Investigation of heterogeneity in reported effects	State the method(s) used to examine heterogeneity in reported effects when it was not possible to undertake a meta-analysis of effect estimates and its extensions to investigate heterogeneity	4	-
**6** Certainty of evidence	Describe the methods used to assess certainty of the synthesis findings	4	-
**7** Data presentation methods	Describe the graphical and tabular methods used to present the effects (e.g., tables, forest plots, harvest plots).<br>Specify key study characteristics (e.g., study design, risk of bias) used to order the studies, in the text and any tables or graphs, clearly referencing the studies included	-	-
Results			
**8** Reporting results	For each comparison and outcome, provide a description of the synthesised findings, and the certainty of the findings. Describe the result in language that is consistent with the question the synthesis addresses, and indicate which studies contribute to the synthesis	4-12	-
Discussion			
**9** Limitations of the synthesis	Report the limitations of the synthesis methods used and/or the groupings used in the synthesis, and how these affect the conclusions that can be drawn in relation to the original review question	13-15	-

Supplementary Table S3: List of full-text escluded.

Title	Authors	Pubblication year	Reason for exclusion
MicroRNA-218 inhibits tumor angiogenesis of human renal cell carcinoma by targeting GAB2.	Mu, Lijun;Guan, Bing;Tian, Juanhua;Li, Xiang;Long, Qingzhi;Wang, Meiyu;Wang, Wen;She, Junjun;Li, Xudong;Wu, Dapeng;Du, Yuefeng	2020	Wrong outcome
Chasing the Role of miRNAs in RCC: From Free-Circulating to Extracellular-Vesicle-Derived Biomarkers.	Mastrolia, Ilenia;Catani, Virginia;Oltrecolli, Marco;Pipitone, Stefania;Vitale, Maria Giuseppa;Masciale, Valentina;Chiavelli, Chiara;Bortolotti, Carlo Augusto;Nasso, Cecilia;Grisendi, Giulia;Sabbatini, Roberto;Dominici, Massimo	2023	Wrong study design
Comprehensive Analysis of Transcriptional Expression of hsa-mir-21 Predicted Target Genes and Immune Characteristics in Kidney Renal Clear Cell Carcinoma.	Xu, Da-Ming;Li, Ming;Lin, Shu-Bin;Yang, Zheng-Liang;Xu, Teng-Yu;Yang, Jin-Huan;Yin, Jun	2022	Wrong study design
Comprehensive analysis of PTEN-related ceRNA network revealing the key pathways WDFY3-AS2 - miR-21-5p/miR-221-3p/miR-222-3p - TIMP3 as potential biomarker in tumorigenesis and prognosis of kidney renal clear cell carcinoma.	Zhou, Xishan;Liu, Guofeng;Xu, Mo;Ying, Xintao;Li, Bianfeng;Cao, Fengxi;Cheng, Shuqiang;Xiao, Beibei;Cheng, Miao;Liang, Liang;Jia, Mingxi;Li, Wen;Liu, Jiheng;Li, Zheng	2022	Not retrieved
The translational potential of microRNAs as biofluid markers of urological tumours.	Fendler, Annika;Stephan, Carsten;Yousef, George M;Kristiansen, Glen;Jung, Klaus	2016	Wrong study design
Control of Angiogenesis via a VHL/miR-212/132 Axis.	Lei, Zhiyong;Klasson, Timothy D;Brandt, Maarten M;van de Hoek, Glenn;Logister, Ive;Cheng, Caroline;Doevendans, Pieter A;Sluijter, Joost P G;Giles, Rachel H	2020	Wrong outcome
Small extracellular vesicles as a multicomponent biomarker platform in urinary tract carcinomas.	K, Szeliski;T, Drewa;M, Pokrywczyńska	2022	Wrong study design
LncRNA ADAMTS9-AS2 inhibits cell proliferation and decreases chemoresistance in clear cell renal cell carcinoma via the miR-27a-3p/FOXO1 axis.	Song, Er-Lin;Xing, Li;Wang, Liang;Song, Wen-Ting;Li, Dan-Bin;Wang, Yi;Gu, Yi-Wei;Liu, Ming-Ming;Ni, Wen-Jun;Zhang, Peng;Ma, Xin;Zhang, Xu;Yao, Jie;Chen, Yang;An, Rui-Hua	2019	Wrong outcome
Identification of angiogenesis-related miRNAs in a population of patients with renal clear cell carcinoma.	Li, He-Cheng;Li, Jian-Ping;Wang, Zi-Ming;Fu, De-Lai;Li, Zhao-Lun;Zhang, Dong;Gan, Wei-Min;Chong, Tie	2014	Wrong outcome
Proteins S100A8 and S100A9 are potential biomarkers for renal cell carcinoma in the early stages: results from a proteomic study integrated with bioinformatics analysis.	Zhang, Limin;Jiang, Haowen;Xu, Gang;Wen, Hui;Gu, Bin;Liu, Jun;Mao, Shanghua;Na, Rong;Jing, Yan;Ding, Qiang;Zhang, Yuanfang	2015	Wrong outcome
Dissecting order amidst chaos of programmed cell deaths: construction of a diagnostic model for KIRC using transcriptomic information in blood-derived exosomes and single-cell	Wang, Chengbang;He, Yuan;Zheng, Jie;Wang, Xiang;Chen, Shaohua	2023	Wrong outcome

multi-omics data in tumor microenvironment.			
Exosome-Transmitted lncARSR Promotes Sunitinib Resistance in Renal Cancer by Acting as a Competing Endogenous RNA.	Qu, Le;Ding, Jin;Chen, Cheng;Wu, Zhen-Jie;Liu, Bing;Gao, Yi;Chen, Wei;Liu, Feng;Sun, Wen;Li, Xiao-Feng;Wang, Xue;Wang, Yue;Xu, Zhen-Yu;Gao, Li;Yang, Qing;Xu, Bin;Li, Yao-Ming;Fang, Zi-Yu;Xu, Zhi-Peng;Bao, Yi;Wu, Deng-Shuang;Miao, Xiong;Sun, Hai-Yang;Sun, Ying-Hao;Wang, Hong-Yang;Wang, Lin-Hui	2016	Wrong outcome
Exosomal microRNA-1 and MYO15A as a target for therapy and diagnosis in renal cell carcinoma.	Yoshino, Hirofumi;Tatarano, Shuichi;Tamai, Motoki;Tsuruda, Masafumi;Iizasa, Sayaka;Arima, Junya;Kawakami, Issei;Fukumoto, Wataru;Kawahara, Ichiro;Li, Gang;Sakaguchi, Takashi;Inoguchi, Satoru;Yamada, Yasutoshi;Enokida, Hideki	2022	Wrong outcome
Everolimus resistance in clear cell renal cell carcinoma: miRNA-101 and HIF-2α as molecular triggers?	Nogueira, Inês;Dias, Francisca;Morais, Mariana;Teixeira, Ana Luísa;Medeiros, Rui	2019	Wrong outcome
Expression of serum microRNA-378 and its clinical significance in renal cell carcinoma.	Shi, Lixin;Zhang, Lei;Wang, Chunyang;Sun, Shengkun;Cao, Xiyuan;Zhang, Xu	2017	Retired article
Profiling of Serum Extracellular Vesicles Reveals miRNA-4525 as a Potential Biomarker for Advanced Renal Cell Carcinoma.	Muramatsu-Maekawa, Yuka;Kawakami, Kyojiro;Fujita, Yasunori;Takai, Manabu;Kato, Daiki;Nakane, Keita;Kato, Taku;Tsuchiya, Tomohiro;Koie, Takuya;Miura, Yuri;Ito, Masafumi;Mizutani, Kosuke	2021	Wrong outcome
Construction and validation of a novel ten miRNA-pair based signature for the prognosis of clear cell renal cell carcinoma.	Wang, Yulin;Shen, Ziyan;Mo, Shaocong;Dai, Leijie;Song, Biao;Gu, Wenchao;Ding, Xiaoqiang;Zhang, Xiaoyan	2022	Wrong outcome
Identification of dysregulated serum miR-508-3p and miR-885-5p as potential diagnostic biomarkers of clear cell renal carcinoma.	Liu, Siming;Deng, Xiaojun;Zhang, Jiong	2019	Wrong comparator
MicroRNA-204-5p Hampers the Malignant Progression of Clear Cell Renal Cell Carcinoma through GXYLT2 Downregulation.	Wu, Ying;Liao, Qin	2022	Wrong outcome
circTLK1 facilitates the proliferation and metastasis of renal cell carcinoma by regulating miR-495-3p/CBL axis.	Lei, Xiangli;Yang, Meiling;Xiao, Zhifang;Zhang, Heng;Tan, Shuai	2021	Wrong outcome
MiR429 expression level in renal cell cancer and its correlation with the prognosis of patients.	Wang, Zhan-Kun;Luo, Lei;Du, Zhao-Jin;Zhang, Gui-Ming;Sun, Li-Jiang	2017	Wrong outcome
Differential microRNA expression in renal cell carcinoma.	Cheng, Tingting;Wang, Lina;Li, Yuyao;Huang, Chen;Zeng, Lingxia;Yang, Jin	2013	Wrong comparator
Circulating microRNAs from the Molecular Mechanisms to Clinical Biomarkers: A Focus on the Clear Cell Renal Cell Carcinoma.	Tito, Claudia;De Falco, Elena;Rosa, Paolo;Iaiza, Alessia;Fazi, Francesco;Petrozza, Vincenzo;Calogero, Antonella	2021	Wrong study design

MiR-193b inhibits the growth and metastasis of renal cell carcinoma by targeting IGF1R.	Chen, Jianhui;Deng, Ting;Li, Xiaofan;Cai, Weizhong	2019	Wrong outcome
MicroRNA Derived from Circulating Exosomes as Noninvasive Biomarkers for Diagnosing Renal Cell Carcinoma.	Xiao, Chu-Tian;Lai, Wen-Jie;Zhu, Wei-An;Wang, Hua	2020	Wrong comparator
MiR-483-5p downregulation contributed to cell proliferation, metastasis, and inflammation of clear cell renal cell carcinoma.	Wang, Xue-Gang;Zhu, Yong-Wu;Wang, Tao;Chen, Bin;Xing, Jin-Chun;Xiao, Wen	2021	Wrong comparator
The prognostic value of miR-487a in clear cell renal cell carcinoma and its influence on cell biological behavior.	Liu, Shuzhen;Zhao, Yuling;Zhao, Yubin;Tang, Xiaochun	2022	Wrong outcome
Exosomal MicroRNA Levels Associated with Immune Checkpoint Inhibitor Therapy in Clear Cell Renal Cell Carcinoma.	Ivanova, Elizaveta;Asadullina, Dilara;Gilyazova, Gulshat;Rakhimov, Radmir;Izmailov, Adel;Pavlov, Valentin;Khusnutdinova, Elza;Gilyazova, Irina	2023	Wrong outcome
Serum exosomal miR-210 as a potential biomarker for clear cell renal cell carcinoma.	Wang, Xuegang;Wang, Tao;Chen, Chenxi;Wu, Zhun;Bai, Peide;Li, Shouchun;Chen, Bin;Liu, Rongfu;Zhang, Kaiyan;Li, Wei;Chen, Yuedong;Xing, Jinchun	2019	Wrong outcome
Serum extracellular vesicles derived hsa-miR-320d as an indicator for progression of clear cell renal cell carcinoma.	Xue, Yizheng;Chen, Tianyi;Hou, Naiqiao;Wu, Xiaorong;Kong, Wen;Huang, Jiwei;Zhang, Jin;Chen, Yonghui;Zheng, Junhua;Zhai, Wei;Xue, Wei	2023	Wrong outcome
Circ_0037866 Contributes to the Tumorigenesis of Renal Cell Carcinoma by Sequestering miR-384 to Elevate Chromobox 5 Expression.	Shi, Xiaoqiang;Song, Shichao;Gao, Ying;Cui, Zhenyu;Wang, Wentao;Liu, Mingkai	2022	Wrong outcome
Serum miR-210 as a potential biomarker of early clear cell renal cell carcinoma.	Iwamoto, Hideto;Kanda, Yusuke;Sejima, Takehiro;Osaki, Mitsuhiko;Okada, Futoshi;Takenaka, Atsushi	2014	Wrong outcome
[Correlation between serum level of miRNA-106a expression with clinicopathological characteristics and prognosis of patients with renal cell carcinoma].	Yang, Qingyan;Liu, Junyi;Liang, Yalin;Wang, Changan;Han, Jianle;Zhu, Litao;Yuan, Shengping;Sun, Qiang;Zhang, Hongsen	2021	Not retrieved
Feedback activation of GATA1/miR-885-5p/PLIN3 pathway decreases sunitinib sensitivity in clear cell renal cell carcinoma.	Yao, Dayong;Xia, Shunyao;Jin, Chengjun;Zhao, Weiming;Lan, Wenjia;Liu, Zan;Xiu, Youcheng	2020	Wrong outcome
MiR-765 functions as a tumour suppressor and eliminates lipids in clear cell renal cell carcinoma by downregulating PLP2.	Xiao, Wen;Wang, Cheng;Chen, Ke;Wang, Tao;Xing, Jinchun;Zhang, Xiaoping;Wang, Xuegang	2020	Wrong comparator
Identification of aberrant tRNA-halves expression patterns in clear cell renal cell carcinoma.	Nientiedt, Malin;Deng, Mario;Schmidt, Doris;Perner, Sven;Müller, Stefan C;Ellinger, Jörg	2016	Wrong outcome

Combination of the histone deacetylase inhibitor vorinostat with bevacizumab in patients with clear-cell renal cell carcinoma: a multicentre, single-arm phase I/II clinical trial.	Pili, Roberto;Liu, Glenn;Chintala, Sreenivasulu;Verheul, Hendrick;Rehman, Shabnam;Attwood, Kristopher;Lodge, Martin A;Wahl, Richard;Martin, James I;Miles, Kiersten Marie;Paesante, Silvia;Adelaiye, Remi;Godoy, Alejandro;King, Serina;Zwiebel, James;Carducci, Michael A	2017	Wrong outcome
[Detection of cell-free lncRNA in serum of cancer patients].	Kohls, K;Schmidt, D;Holdenrieder, S;Müller, S C;Ellinger, J	2015	No english language
High serum miR-183 level is associated with poor responsiveness of renal cancer to natural killer cells.	Zhang, Qunmei;Di, Wenyu;Dong, Yuqian;Lu, Guangjian;Yu, Jian;Li, Jinsong;Li, Pingfa	2015	Wrong outcome
miR-29b and miR-198 overexpression in CD8+ T cells of renal cell carcinoma patients down-modulates JAK3 and MCL-1 leading to immune dysfunction.	Gigante, Margherita;Pontrelli, Paola;Herr, Wolfgang;Gigante, Maddalena;D'Avenia, Morena;Zaza, Gianluigi;Cavalcanti, Elisabetta;Accetturo, Matteo;Lucarelli, Giuseppe;Carrieri, Giuseppe;Battaglia, Michele;Storkus, Walter J;Gesualdo, Loreto;Ranieri, Elena	2016	Wrong outcome
Novel potential predictive markers of sunitinib outcomes in long-term responders versus primary refractory patients with metastatic clear-cell renal cell carcinoma.	Puente, Javier;Laínez, Nuria;Dueñas, Marta;Méndez-Vidal, María José;Esteban, Emilio;Castellano, Daniel;Martinez-Fernández, Mónica;Basterretxea, Laura;Juan-Fita, María José;Antón, Luis;León, Luis;Lambea, Julio;Pérez-Valderrama, Begoña;Vázquez, Sergio;Suarez, Cristina;Del Muro, Xavier Garcia;Gallardo, Enrique;Maroto, José Pablo;Samaniego, M Luz;Suárez-Paniagua, Beatriz;Sanz, Julián;Paramio, Jesús M	2017	Wrong outcome
Let-7d suppresses growth, metastasis, and tumor macrophage infiltration in renal cell carcinoma by targeting COL3A1 and CCL7.	Su, Boxing;Zhao, Wei;Shi, Bentao;Zhang, Zhongyuan;Yu, Xi;Xie, Feng;Guo, Zhongqiang;Zhang, Xiaoyu;Liu, Jin;Shen, Qi;Wang, Jinghua;Li, Xuesong;Zhang, Zhiqian;Zhou, Liqun	2014	Wrong outcome
Phase I study of the mTOR inhibitor everolimus in combination with the histone deacetylase inhibitor panobinostat in patients with advanced clear cell renal cell carcinoma.	Wood, Anthony;George, Saby;Adra, Nabil;Chintala, Sreenivasulu;Damayanti, Nur;Pili, Roberto	2020	Wrong outcome
Let-7d inhibits intratumoral macrophage M2 polarization and subsequent tumor angiogenesis by targeting IL-13 and IL-10.	Su, Boxing;Han, Haibo;Gong, Yanqing;Li, Xuesong;Ji, Chaoyue;Yao, Jingjing;Yang, Jianghui;Hu, Weiguo;Zhao, Wei;Li, Jianxing;Zhang, Gang;Zhou, Liqun	2021	Wrong outcome
Down-regulation of BTG1 by miR-454-3p enhances cellular radiosensitivity in renal carcinoma cells.	Wu, Xin;Ding, Nan;Hu, Wentao;He, Jinpeng;Xu, Shuai;Pei, Hailong;Hua, Junrui;Zhou, Guangming;Wang, Jufang	2014	Wrong outcome
miRConnect 2.0: identification of oncogenic, antagonistic miRNA families in three human cancers.	Hua, Youjia;Larsen, Niels;Kalyana-Sundaram, Shanker;Kjems, Jørgen;Chinnaiyan, Arul M;Peter, Marcus E	2013	Wrong study design

A miR-192-EGR1-HOXB9 regulatory network controls the angiogenic switch in cancer.	Wu, Sherry Y;Rupaimoole, Rajesha;Shen, Fangrong;Pradeep, Sunila;Pecot, Chad V;Ivan, Cristina;Nagaraja, Archana S;Gharpure, Kshipra M;Pham, Elizabeth;Hatakeyama, Hiroto;McGuire, Michael H;Haemmerle, Monika;Vidal-Anaya, Viviana;Olsen, Courtney;Rodriguez-Aguayo, Cristian;Filant, Justyna;Ehsanipour, Ehsan A;Herbrich, Shelley M;Maiti, Sourindra N;Huang, Li;Kim, Ji Hoon;Zhang, Xinna;Han, Hee-Dong;Armaiz-Pena, Guillermo N;Seviour, Elena G;Tucker, Sue;Zhang, Min;Yang, Da;Cooper, Laurence J N;Ali-Fehmi, Rouba;Bar-Eli, Menashe;Lee, Ju-Seog;Ram, Prahlad T;Baggerly, Keith A;Lopez-Berestein, Gabriel;Hung, Mien-Chie;Sood, Anil K	2016	Wrong outcome
The Axl-Regulating Tumor Suppressor miR-34a Is Increased in ccRCC but Does Not Correlate with Axl mRNA or Axl Protein Levels.	Fritz, Helena K;Gustafsson, Anna;Ljungberg, Börje;Ceder, Yvonne;Axelson, Håkan;Dahlbäck, Björn	2015	Wrong outcome
Evaluation of reference genes for the analysis of serum miRNA in patients with prostate cancer, bladder cancer and renal cell carcinoma.	Sanders, Imke;Holdenrieder, Stefan;Walgenbach-Brünagel, Gisela;von Ruecker, Alexander;Kristiansen, Glen;Müller, Stefan C;Ellinger, Jörg	2012	Wrong outcome
MicroRNA expression profiling of peripheral blood samples predicts resistance to first-line sunitinib in advanced renal cell carcinoma patients.	Gámez-Pozo, Angelo;Antón-Aparicio, Luis M;Bayona, Cristina;Borrega, Pablo;Gallegos Sancho, María I;García-Domínguez, Rocío;de Portugal, Teresa;Ramos-Vázquez, Manuel;Pérez-Carrión, Ramón;Bolós, María V;Madero, Rosario;Sánchez-Navarro, Iker;Fresno Vara, Juan A;Espinosa Arranz, Enrique	2012	Wrong outcome
Tumour angiogenesis regulation by the miR-200 family.	Pecot, Chad V;Rupaimoole, Rajesha;Yang, Da;Akbani, Rehan;Ivan, Cristina;Lu, Chunhua;Wu, Sherry;Han, Hee-Dong;Shah, Maitri Y;Rodriguez-Aguayo, Cristian;Bottsford-Miller, Justin;Liu, Yuexin;Kim, Sang Bae;Unruh, Anna;Gonzalez-Villasana, Vianey;Huang, Li;Zand, Behrouz;Moreno-Smith, Myrthala;Mangala, Lingegowda S;Taylor, Morgan;Dalton, Heather J;Sehgal, Vasudha;Wen, Yunfei;Kang, Yu;Baggerly, Keith A;Lee, Ju-Seog;Ram, Prahlad T;Ravoori, Murali K;Kundra, Vikas;Zhang, Xinna;Ali-Fehmi, Rouba;Gonzalez-Angulo, Ana-Maria;Massion, Pierre P;Calin, George A;Lopez-Berestein, Gabriel;Zhang, Wei;Sood, Anil K	2013	Wrong study design

Non-coding RNAs in cancer brain metastasis.	Wu, Kerui;Sharma, Sambad;Venkat, Suresh;Liu, Keqin;Zhou, Xiaobo;Watabe, Kounosuke	2016	Wrong study design
Advances in Brain Metastases Diagnosis: Non-coding RNAs As Potential Biomarkers.	Eraky, Akram M	2023	Wrong study design
MicroRNA-196a2 Biomarker and Targetome Network Analysis in Solid Tumors.	Toraih, Eman A;Fawzy, Manal S;Mohammed, Eman A;Hussein, Mohammad H;El-Labban, Mohamad M	2016	Not retrieved

Supplementary Table S4: Laboratory findings regarding extraction, normalization and quantitative analysis of selected studies. NR: not reported.

**Study (first author, publication year)**	**Tools for RNA extraction**	**Normalized used**	**Mehtod for quantitative analysis of miRNAs expression**
**Chanudet E. (2017)**	NucleoSpin® miRNA Plasma kit	Endogenous (U6snRNA, let7d, let7g and let7i)	TaqMan Human MicroRNA Array A + B Card Set v3.0 (Applied Biosystems, Foster City, CA) and ABI 7900HT SDS software v2.4. Cycle threshold (Ct)
**Chen X. (2021)**	TRIzolTM LS Reagent (Invitrogen, USA) and NanoDrop 2000c system (Thermo Scientific, USA)	Synthetic (cel-miR-39)	LightCycler 480 Real-Time PCR System (Roche Diagnostics, Mannheim, Germany) and SYBR Green qPCR kit (SYBR Pre-mix Ex Taq II, TaKaRa)
**Fedorko M. (2015)**	Qiagen miRNeasy Mini Kit (Qiagen, GmbH, Hilden, Germany) and NanoDrop ND-1000 Spectrophotometer (Thermo Scientific, Wilmington, DE, USA)	NR	TaqMan MicroRNA Assays (Applied Biosystems, Carlsbad, CA, USA) following reverse transcription (TaqMan MicroRNA Reverse Transcription Kit, Applied Biosystems)
**Hauser S. (2012)**	mirVana PARIS Kit (Ambion, Foster City, CA,USA)	Synthetic (cel-miR-39)	TaqMan Small RNA Assay on the ABIPrism 7900HT
**Heinemann F. G. (2018)**	QiagenmiR Neasy Serum/Plasma kit (Hilden, Germany)	Endogenous (miR-16, miR-191-5p, and miR-320a)	Qiagen miScript SYBR Green PCR Kit on an Ap plied Biosystems 7900 HT Fast Real-Time PCR System (Thermo Fisher Scientific, Waltham, USA) and Illumina NextSeq 500 sequencer (SanDiego, USA)
**Huang G. (06/2020)**	TRIzolTM LS Reagent (Invitrogen, USA) and NanoDrop 2000c system (Thermo Scientific, USA)	Synthetic (cel-miR-39)	SYBRGreenqPCRkit (SYBRPre-mixExTaq II, TaKaRa) on LightCycler 480 Real-Time PCR System (Roche Diagnostics, Mannheim, Germany)

**Huang G. (07/2020)**	TRIzolTM LS Reagent (Invitrogen, USA) and NanoDrop 2000c system (Thermo Scientific, USA)	Synthetic (cel-miR-39)	SYBRGreenqPCRkit (SYBRPre-mixExTaq II, TaKaRa) on LightCycler 480 Real-Time PCR System (Roche Diagnostics, Mannheim, Germany)
**Iwamoto H. (2014)**	mirVana™ miRNA isolation kit (Ambion, USA)	Endogenous (miR-16)	TaqMan MicroRNA assay on the ABI PRISM 7900HT system (Applied Biosystems)
**Kalogirou C. (2020)**	Exiqon RNA services	Endogenous (miR-451a-5p)	NR
**Li M. (2017)**	Trizol reagent (Invitrogen, Carlsbad, CA, USA)	Endogenous (U6-snRNA)	SYBR GREEN PCR Master Mix (Takala, Dalian, China) and analyzed with an ABI7500 Real-time PCR system (Applied Biosystems)
**Li R. (2022)**	TRIzol LS isolation kit (Thermo Fisher Scientific, Waltham, MA, UnitedStates) and NanoDrop 2000c spectrophotometer (Thermo Scientific, United States)	Synthetic (cel-miR-54-5p)	LightCycler480 Real-Time PCR system (Roche Diagnostics, Mannheim, Germany)
**Li R. (2023)**	TRIzol LS isolation kit(Thermo Fisher Scientific, Waltham, MA, USA) and NanoDrop 2000 spectrophotometer (NanoDrop, Wilmington, DE, USA)	NR	Taqman probe on LightCycler 480 Real TimePCR System (RocheDiagnostics, Mannheim, Germany)
**Liu T.Y. (2015)**	Trizol (Invitrogen, Carlsbad, CA, USA) and ultraviolet spectromethron	NR	7900 Type Real-Time PCR instrument (Applied Biosystems, Foster City, CA, USA)
**Liu Z. (2021)**	Trizol reagent (Invitrogen, USA)	Endogenoou (U6-snRNA)	RT-PCR kit (TransGen, China)
**Lou N. (2016)**	TRI Reagent BD (Molecular Research Center, Cincinnati, USA) and NanoDrop 2000 spectrophotometer (NanoDrop Technologies, Wilmington, USA)	Endogenous (RNU6B (U6), miR-320c) and Synthetic (cel-miR-39)	LightCycler 480II; Roche, Basel, Switzerland) and SYBR Green mix (Thermo, Massachusetts, USA)
**Redova M.**	Qiagenmi Rneasy MiniKit (Qiagen, GmbH, Germany) and NanoDrop ND 1000 Spectrophotometer (ThermoScientific, Wilmington, DE, USA)	Endogenous (miR-16)	TaqMan MicroRNA Reverse Transcription Kit and Megaplex RT setpool A and B version 2.0 (Applied Biosystems, CA, USA) and ABI 7900HT Instrument (Applied Biosystems, CA, USA)

**Texeira A. L. (2013)**	mirVana™ PARIS™ Kit (Ambion®)	Endogenous (RNU44)	TaqMan® MicroRNA Reverse Transcription Kit (Applied Biosystems®) and StepOne Software v2.2 (Applied Biosystems®)
**Texeira A. L. (2014)**	QIAamp DNA Minikit (Qiagen)	Endogenous (RNU48)	TaqMan® MicroRNA Reverse Transcription Kit (Applied Biosystems®) and StepOne Software v2.2 (Applied Biosystems®)
**Tusong H. (2016)**	mirVana PARIS Kit (Ambion, Austin, TX, USA)	Endogenous (U6-snRNA)	Maxima SYBR Green qPCR Kit (Thermo Scientific)
**Wang C. (2014)**	TRIzol reagent (Invitrogen, Carlsbad, CA, USA)	Endogenous (combination of let-7d, let-7g and let-7i)	
**Wang X. (2016)**	TRIzol reagent (Invitrogen)	NR	TaqMan MicroRNA assays (Applied Biosystems, Foster City, CA, USA) and SyBR green PCR master mix (Applied Biosystems)
**Wen Z. (2023)**	TRIzol LS isolation kit (Invitrogen, USA) and NanoDrop 2000c (Thermofisher, USA)	Synthetic (cel-miR-54)	LightCycler 480 Real-Time PCR System (Roche Diagnostics, Germany) and the TaqMan probe (Sangon, China)
**Wulfken L. M.**	mirVana PARIS Kit (Ambion, Foster City, CA, USA)	Synthetic (cel-miR-39)	TaqMan Small RNA Assay and the ABI Prism 7900HT
**Yadav S. (2017)**	miRNeasy mini kit (Qiagen) and miRNA Serum/ Plasma kit (Qiagen)	Synthetic (cel-miR-39)	miScript SYBR Green PCR Kit (Qiagen)
**Zhang Q. (2015)**	TRIzol solution (Invitrogen, Carlsbad, CA, USA)	NR	SYBR Premix Ex Taq II kit (Takara, Japan) on an ABI-7300 Real-Time PCR System (Applied Biosystems, Foster City, CA, USA)
**Zhao A. (2012)**	MicroMini Kit (Qiagen) and Nanodrop ND-1000 (Thermo Scientific)	Endogenous (5srRNA)	miScript SYBR Green PCR kit (Qiagen)
